# ppdb: plant promoter database version 3.0

**DOI:** 10.1093/nar/gkt1027

**Published:** 2013-11-03

**Authors:** Ayaka Hieno, Hushna Ara Naznin, Mitsuro Hyakumachi, Tetsuya Sakurai, Mututomo Tokizawa, Hiroyuki Koyama, Naoki Sato, Tomoaki Nishiyama, Mitsuyasu Hasebe, Andreas D. Zimmer, Daniel Lang, Ralf Reski, Stefan A. Rensing, Junichi Obokata, Yoshiharu Y. Yamamoto

**Affiliations:** ^1^The United Graduate School of Agricultural Science, Gifu University, 1-1 Yanagido, Gifu City, Gifu 501-1193 Japan, ^2^Faculty of Applied Biological Sciences, Gifu University, 1-1 Yanagido, Gifu City, Gifu 501-1193 Japan, ^3^Center for Sustainable Resource Science, RIKEN Yokohama Institute, 1-7-22 Suehiro-cho, Tsurumi-ku, Yokohama, Kanagawa 230-0045 Japan, ^4^Graduate School of Arts and Sciences, The University of Tokyo, 3-8-1 Komaba, Meguro-ku, Tokyo 153-8902 Japan, ^5^Advanced Science Research Center, Kanazawa University, Takaramachi 13-1. Kanazawa City, Ishikawa 920-0934, Japan, ^6^National Institute for Basic Biology, Nishigonaka 38, Myodaiji, Okazaki City, Aichi 444-8585 Japan, ^7^Department of Basic Biology, School of Life Science, Graduate University for Advanced Studies, Okazaki 444-8585, Japan, ^8^Faculty of Biology, University of Freiburg, Schänzlestrasse 1, D79104 Freiburg, Germany, ^9^Plant Cell Biology, Faculty of Biology, University of Marburg, Karl-von-Frisch-Strasse 9, 35043 Marburg, Germany and ^10^Graduate School of Environmental Life Science, Kyoto Prefectural University, 1-5 Hangi-cho, Shimogamo, Sakyo-ku, Kyoto 606-8522 Japan

## Abstract

ppdb (http://ppdb.agr.gifu-u.ac.jp) is a plant promoter database that provides information on transcription start sites (TSSs), core promoter structure (TATA boxes, Initiators, Y Patches, GA and CA elements) and regulatory element groups (REGs) as putative and comprehensive transcriptional regulatory elements. Since the last report in this journal, the database has been updated in three areas to version 3.0. First, new genomes have been included in the database, and now ppdb provides information on *Arabidopsis thaliana*, rice, *Physcomitrella patens* and poplar. Second, new TSS tag data (34 million) from *A. thaliana*, determined by a high throughput sequencer, has been added to give a ∼200-fold increase in TSS data compared with version 1.0. This results in a much higher coverage of ∼27 000 *A. thaliana* genes and finer positioning of promoters even for genes with low expression levels. Third, microarray data-based predictions have been appended as REG annotations which inform their putative physiological roles.

## INTRODUCTION

Gene regulation is a central part of morphogenesis and environmental adaptation of higher plants, and it is controlled by the promoter of each gene. Therefore, understanding of promoter structure is crucial to understand these fundamental processes of plants.

There are three aspects to promoter structure: (i) the position, direction and strength of the transcription start sites (TSSs) that indicate actual promoter position; (ii) the type and position of the core promoter elements such as TATA boxes and Initiators (Inrs) that are thought to be the major determinants of the direction and position of promoters and (iii) the type and position of transcriptional regulatory elements that are involved in gene regulation.

In our last report (1), we introduced the plant promoter database (ppdb), which provided promoter information about TSS clusters, core promoter elements [TATA boxes, Inrs, Y Patches, GA and CA elements (2,3)] and regulatory element groups [REGs, putative position-sensitive transcriptional regulatory elements that are extracted by local distribution of short sequences (LDSS) analysis (2)] as putative and comprehensive sets of transcriptional regulatory elements. The database of the original version 1.0 contained information of two plant species, *Arabidopsis thaliana* and rice.

## MAJOR EXTENSIONS FROM VERSION 1.0

The major amendment in version 3.0 is the addition of the *Physcomitrella patens* and poplar genomes to the database. The sources used for the information of the four genomes, including *A. thaliana* and rice, are shown in Table 1. The promoter elements of the moss genome have been extracted by the LDSS method (2). During extraction, we noticed that considerable numbers of moss genes are driven by a similar type of promoter that is located within long terminal repeats. These promoters affect the extraction process due to tight sequence conservation that is not related to promoter function and for this reason they were excluded from the LDSS analysis. *A. thaliana* promoter elements have been applied to the poplar genome because the Brassicaceae and Malpighiales are phylogenetically close.

**Table 1.** Source of ppdb version 3.0

	Specification	Source	Size
*A. thaliana*			
Genome sequence and gene annotation	TAIR9	http://www.arabidopsis.org/, (4)	
TSS information	Selected RAFL cDNA	http://rarge.gsc.riken.jp/, (5)	62 108 (clones^a^)
	Cap signature CT-MPSS tags	(3)	158 237 (tags^b^)
	Oligo-Cap Illumina data	Tokizawa M, Yamanaka H, Koyama H, Sakurai T, Kurotani A, Shinozaki K, Suzuki Y, Sugano S, Obokata J, Yamamoto YY (unpublished data)	34 206 936 (tags^b^)
Promoter elements	*A. thaliana* LDSS-positive octamers	(2,3)	659 (octamers^c^)
	Annotation for LDSS elements: PLACE	http://www.dna.affrc.go.jp/PLACE/, (6)	21 (only matched motifs^d^)
	Annotation for LDSS elements: stress and hormonal responses	(7)	53 (only matched motifs^c^)
Rice (*Oryza sativa*)			
Genome sequence and gene annotation	RGSP build 4.0	http://rapdb.lab.nig.ac.jp/, (8)	—
TSS information	Carefully selected fl-cDNA (from KOME)	http://cdna01.dna.affrc.go.jp/cDNA/, (9)	17 286 (clones^a^)
Promoter elements	Rice LDSS-positive octamers	(2,10)	660 (octamers^c^)
	Annotation for LDSS elements: PLACE	http://www.dna.affrc.go.jp/PLACE/, (6)	4 (only matched motifs^d^)
Moss (*P. patens*)			
Genome sequence and gene annotation	JGI version 1.1, COSMOSS V1.6	http://www.cosmos.org, (11,12)	—
TSS information	5′ CAGE	(13)	1 122 382 (tags^b^)
Promoter elements	*P. patens* LDSS-positive octamers	This work	198 (octamers^c^)
Poplar (*Populus trichocarpa*)			
Genome sequence and gene annotation	Phytozome6	http://www.phytozome.net/poplar, (14)	—
TSS information	FL-cDNA info from GenBank	(15)	15 256 (clones^a^, BP921855–937111)
			36 103 (clones^a^, DB874873–910976)
Promoter elements	*A. thaliana* LDSS-positive octamers	(2,3)	659 (octamers^c^)
Orthologue gene			
Orthologue group	Gclust	(16)	336 689 (families^e^)

^a^clone number, ^b^tag number, ^c^number of octamer sequences, ^d^number of motifs and ^e^number of orthologue families.

A new function called ‘Homologue Gene Search’ has been added to facilitate the comparison of promoter structures of orthologous genes within a species or between different species. Orthologue groups have been determined by Gclust, a system that classifies orthologues according to the presence or absence of protein motifs (16).

**Figure 1.**
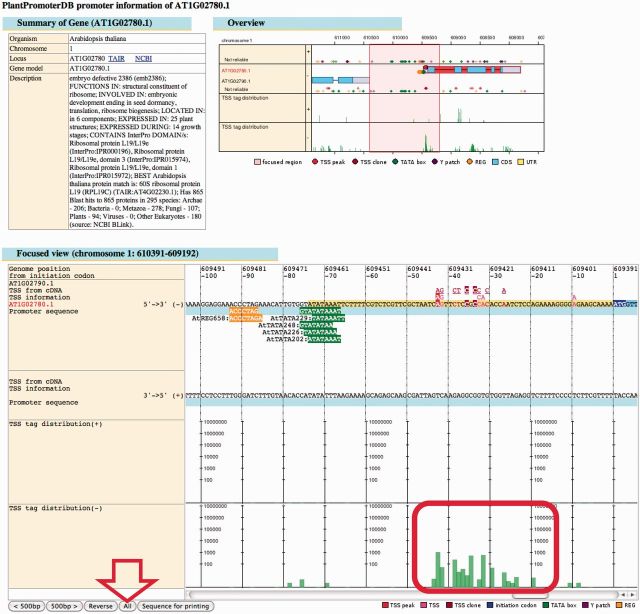
Indication of individual promoters. An *Arabidopsis* gene, AT1G02780.1, is shown. The information is composed of five panels: ‘Summary of Gene’, ‘Overview’, ‘Focused view’ and also ‘Promoter Summary’ (not shown) and ‘Other Reliable Promoter Summary’ (not shown). The top TSS (TSS Peak) is shown in the second column of the ‘Focused view’ as white letters on a red background. New TSS tag data (34 million) are shown at the bottom of ‘Focused view’, highlighted in a red rectangle with rounded corners.

**Figure 2.**
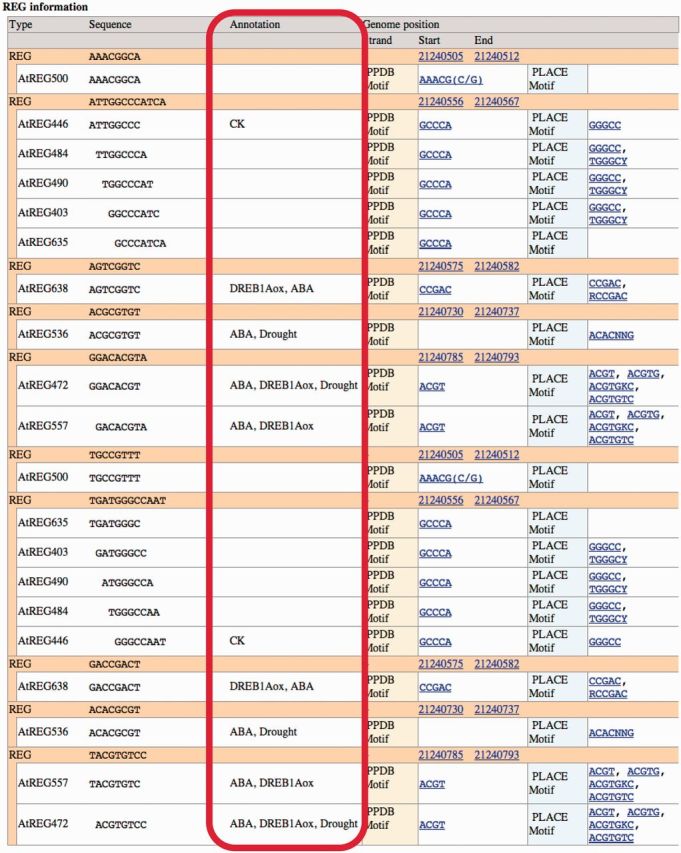
REG information. REG information of the AT5G52310.1 (*RD29A*) promoter is shown. REG annotations, added in version 3.0, are highlighted in a red rectangle with rounded corners.

New *A. thaliana* TSS data of 34 million tags, which corresponds to a ∼200-fold increase in the previous data, have been added (Figure 1). REG annotations have also been appended and show functional predictions based on microarray data of responses to plant hormones (AUX: auxin, BR: brassinosteroid, CK: cytokinin, ABA: abscisic acid, ET: ethylene, JA: jasmonic acid, SA: salicylic acid), responses to a hormone-like chemical (H_2_O_2_) and some environmental stress-related responses (drought, DREB1A overexpression) (7). Functional annotation of 53 of 308 REGs is now available in version 3.0 (Figure 2).

## BROWSING PROMOTER STRUCTURE

The major function of ppdb is to give an indication of a possible promoter structure for each gene in a genome based on the established lists of LDSS-positive elements. The information can be directly called by the gene ID (e.g. AT1G67090 or Os01g0791600), or selected from a list of ‘Keyword Search’ or ‘Homologue Gene Search’. Pages for individual genes show the following information: (i) DNA sequence, (ii) TSS distribution (direction and strength at a 1-bp resolution), (iii) core promoter structure and (iv) REG data.

At the sequence window, promoter elements including REGs and core elements are highlighted in a position-dependent manner as the default setting. Care should be taken that promoters without any TSS information do not show any elements as default. For an indication of the promoter elements of these genes, the ‘Reliable’ button should be clicked which changes the state to ‘All’ (Figure 1, red arrow). This button is a toggle switch between ‘Reliable’ and ‘All’. ‘Reliable’ is a default setting where only elements at appropriate positions relative to the peak TSS are detected. The setting ‘All’ removes the positional restriction as an indication of promoter elements, allowing global detection. The sensitive area in the ‘Reliable’ mode for each element group is described on the front page of the database.

The ‘TSS tag distribution’ columns in the ‘Focused view’ provide the expressional strength of each TSS. The expression is the sum of six TSS tag libraries that are prepared from leaves, roots, inflorescences, etiolated seedlings and shoots from low light-grown and high light-grown seedlings.

The ‘Core promoter information’ table shows the presence or absence of core promoter elements (TATA boxes, Inrs, Y Patches, GA and CA elements).

The ‘REG information’ table shows a REG list together with the corresponding PPDB motifs (2,3) and PLACE motifs (6). REG sequences, as well as PPDB and PLACE motifs, are linked to other pages containing biological information. New REG annotations for *A. thaliana* obtained from predicted *cis*-regulatory elements based on microarray data (7) have been included (Figure 2).

Selection of the ‘All’ button (Figure 1) adds another category, ‘Not Reliable Promoter Summary’ below ‘Other Reliable Promoter Summary’. This category can be used when searching for regulatory elements (REGs) from wider regions or when there is no TSS information on the promoter of interest.

## ADDITIONAL PAGES

A whole list of REGs for each of the genomes can be viewed by selecting a cell in the table of ‘Index of Genes’ at the top of the page. The lists present the relationships between REG ID, sequence, PPDB motifs, PLACE motifs and also functional annotations. Selection of a specific REG entry leads to ‘Summary of the REG’ and ‘Entry Sequences’ that show the whole gene lists containing the corresponding REG, together with gene annotations.

## FUNDING

Grant-in-Aid for Scientific Research on Priority Areas ‘Comparative Genomics’ (in part) (to Y.Y.Y. and J.O.); Scientific Research on Priority Areas ‘Perceptive Plants’ (in part) (to Y.Y.Y.); Grant-in-Aid for Publication of Scientific Research Results ‘Databases’ (in part) (to J.O. and Y.Y.Y.) from the Ministry of Education, Culture, Sports, Science and Technology of Japan; Advanced Low Carbon Technology Research and Development Program from Japan Science and Technology Agency (JST ALCA) (in part) (to Y.Y.Y.). Funding for open access charge: Ministry of Education, Culture, Sports, Science and Technology, Japan.

*Conflict of interest statement.* None declared.
